# Pre-quitting nicotine replacement therapy: Findings from a pilot study

**DOI:** 10.1186/1617-9625-3-2-35

**Published:** 2006-08-15

**Authors:** Chris Bullen, Robyn Whittaker, Natalie Walker, Mark Wallace-Bell

**Affiliations:** 1 Clinical Trials Research Unit, University of Auckland, Auckland, New Zealand; 2 National Addiction Centre, Christchurch School of Medicine, University of Otago, Christchurch, New Zealand

## Abstract

**Background:**

Using nicotine replacement therapy (NRT) while still smoking in the lead up to quitting could enhance success at quitting, one of the most cost-effective means of improving health, but little is known about its acceptability and tolerability.

**Aim:**

To test the acceptability and tolerability of using NRT while smoking for two weeks before quitting, to inform a randomised controlled trial of pre-quitting NRT versus usual NRT-based quitting practice.

**Methods:**

Prospective pragmatic uncontrolled clinic-based pilot study in which 14 adult smokers recruited from a smoking cessation clinic were prescribed nicotine patches or gum with follow up for two weeks. Data were collected on participants' concerns about smoking while using NRT, importance of quitting, urges to smoke, smoking behaviour, previous NRT use and the length of the pre-quitting period. Urine tests were collected weekly for cotinine, and participants recorded smoking activity and noted experiences and changes in their health in diaries.

**Results:**

Only 21% of 14 participants expressed concerns about using NRT while smoking. All of the nine followed up used it as recommended, 56% of these reporting no unpleasant symptoms. Median urine cotinine levels declined over the two weeks. Urges to smoke averaged 1.8 on a 4-point scale. All participants decreased the number of cigarettes per day. Diary records showed wide variation in smoking and NRT use, with an increased sense of control and determination to quit.

**Conclusion:**

Smokers using pre-quitting NRT over two weeks appeared to titrate nicotine levels and symptoms of toxicity were uncommon and of low intensity.

## Introduction

Quitting smoking using nicotine replacement therapy (NRT) is one of the most cost-effective means of reducing risk and improving current and future health, yet sustained quitting rates are still far from ideal.[1] New approaches are needed to enhance the effectiveness of NRT. NRT was licensed to manage tobacco withdrawal symptoms rather than to reduce dependence on cigarette nicotine and so in most Quit programmes NRT is used from the quit date, not before. Two studies suggest positive effects from introducing NRT before the quit date. In a trial of 332 smokers Herrera et al [2] found that allowing participants to familiarize themselves with NRT gum a week before quit day increased abstinence at 6 weeks, though not significantly. Schuurmans et al [3] randomized 200 smokers to receive active or placebo patches two weeks prior to quitting, and showed a near doubling in sustained abstinence at 6 months in the active compared with the control group (22% versus 12%, p = 0.03).

Prior to embarking on a large randomised controlled trial of pre-quitting NRT versus usual practice on quit rates at six months, we undertook a small pilot study involving adult smokers who attended a cessation clinic. We aimed to investigate the acceptability, toxicity and tolerability of NRT while still smoking, for two weeks prior to the nominated quit day.

## Methods

### Participants

Adult smokers of at least 10 cigarettes per day who wanted to quit within the next few weeks were recruited into this prospective, uncontrolled non-experimental study between February–March 2005 at a university cessation clinic in Christchurch, New Zealand in response to a local newspaper advertisement. People with unstable medical conditions, or who were pregnant, breastfeeding or on psychotropic medication, were excluded. The study was approved by the local research ethics committee and all participants provided written informed consent.

### Treatment

Participants received two weeks supply of NRT, either as patches or gum in doses appropriate to their level of dependence, as determined by discussion between client and a cessation therapist (MW-B), to use while still smoking *ad libitum *for two weeks prior to a nominated Quit Day. Instructions were given to participants that they could continue smoking as they wished over this period. Eight weeks of subsidized NRT treatment combined with support telephone calls then followed Quit Day, in line with standard cessation practice in New Zealand. In this pilot study only the two week pre-quit period was studied.

### Measures

Brief questionnaires were administered at enrolment and one week later by one of the investigators (MW-B) to collect demographic (enrolment only) and smoking-related data (including smoking history, cigarette consumption, measure of nicotine dependence and previous NRT use). Participants rated urges to smoke on a 5-point scale (0 = none to 4 = severe), and importance of quitting on a scale of 1 (no importance) to 9 (very important). They provided urine samples for cotinine testing at enrolment, and at seven and fourteen days post-enrolment (Quit Day). Cotinine was measured at a regional hospital laboratory using capillary gas chromatography and nitrogen selective detection. Diaries were given to participants at enrolment to record daily smoking consumption, NRT use and note any toxicity-related symptoms and other relevant experiences. On Quit Day, diaries were collected, final urine cotinine samples taken, a supply of NRT provided for the next 4 weeks and support calls arranged.

## Results

Fourteen of the fifteen (93%) people responding to the newspaper advertisement met the study inclusion criteria, 67% of them female, with a median age of 44 yrs (range 18–70 yrs) and all but one of European New Zealander ethnicity. Participants smoked on average 21 (range 10–35) cigarettes per day (cpd). All were interested in finding new ways to quit, and rated quitting highly (mean 8.4, range 7 – 9). All participants had used NRT before, nine (64%) using patches, two (14%) gum, two (14%) both patch and gum; one did not answer the question. Most (71%) had no concerns about using NRT while they were still smoking. Of those with concerns, two were worried about overdose and the other about feeling light-headed. Eleven (79%) participants kept diaries and completed the follow-up questionnaire

### Pre-quitting NRT use

Eleven participants (79%) used NRT as recommended; three were unresponsive to follow up calls. Eight (64%) received 15 mg/16 hr NRT patch, two (14%) 10 mg/16 hr NRT patch, three (21%) 2 mg NRT gum, and one 4 mg NRT gum. Excerpts from participants' diaries give insight into their experiences as they embarked on the study. Several participants were optimistic about their chances of success:

"I have more success integrating and maintaining new habits when introduced gradually rather than drastic changes" (Start of Day 1, usually smoked 14 cpd)

"Patch on at 7.00 am, coffee and felt that I shouldn't smoke." (Start of Day 1, usually smoked > 30 cpd)

Gum was uniformly regarded as unpleasant to taste whereas patches were well-tolerated. When asked on Day 14 whether the two week period of pre-quitting NRT was adequate to prepare for quitting, 55% preferred at least 3–4 weeks, three felt two weeks was just right while two recommended a shorter period.

### Cigarette consumption and urges to smoke

Considerable day-to-day variation in smoking patterns was evident. Smoking reduced by an average of 4 cpd (range 2–10) in the 11 participants for whom data were available. None stopped smoking completely. Urges to smoke over the pre-quit period were rated mild-moderate, mean score 1.7/4 (range 0–3). Further diary excerpts shed light on the participant's experiences. At the end of the first day, one participant commented on the power of habit despite low level of cravings:

"Didn't often feel like smoking, but habituated ...after lunch...after work" (end of Day 1, usually smoked 20/day)

"Morning craving not so pronounced – cigarettes are beginning to taste awful" (Day 5, usually smoked > 2 cpd)

"Three smokes and one gum at 3.30 pm – feeling confident about working towards quit day" (Day 5, usually smoked 10 cpd)

"I have noticed I am not so preoccupied with making sure I have cigarettes with me when leaving the car" (Day 7, usually smoked 15 cpd)

"For the first time in years did not have cigarette first thing. Felt better. Had three cigarettes later on and felt lousy" (Day 7, usually smoked 15 cpd)

"15 cigarettes today. Started buying 20 packs instead of 30." (Day 13, usually smoked 17–20 cpd)

"Patch on when I awoke; had two cigarettes in the morning, two at midday and one in the afternoon. Felt fine today" (Day 14, usually smoked 30 cpd)

### Symptoms of nicotine toxicity

Participants reported few unpleasant feelings related to use of NRT while smoking. Only four reported experiencing symptoms, including mild nausea, low-grade headaches, sleep disturbance and dizziness, and were most likely due to high nicotine levels rather than withdrawal.[4] Urine cotinine levels converged over the pre-quit period, from a median level of 1515 (range 470–6410) to 1325 (range 470–5210) at two weeks. The subjective experiences documented in the diaries bore no correlation to nicotine levels or with the type or dose of NRT.

"Initially felt 'racy' but [patch] definitely has helped, inhaling less deeply ...able to delay several hours" (Day 14, usually smoked 20 cpd)

## Discussion

Despite being small and uncontrolled, this study nevertheless provides some useful insights into concomitant smoking and NRT use prior to quitting. First, the findings align with those from other studies showing NRT use while smoking to be safe.[5] Few participants experienced symptoms that could be attributed to toxicity, and these were relatively mild in nature. Urine cotinine levels were not at a level to cause concern.

A second and related finding is that urges to smoke were only mild to moderate in strength, and therefore, we suggest, likely to enable a more positive experience for smokers planning to quit compared to the usually abrupt transition. Past positive experiences are the most influential source of self-efficacy, which is important in making behavior changes.[6] Also, easing the trauma of transition around the quit period has been shown to be a significant factor in long term quitting success.[7]

Thirdly, all participants reduced smoking during the pre-quit phase, two thirds by more than 5 cpd, in line with the finding from a review by Fagerstrom and Hughes of eleven studies with concomitant smoking and NRT use in which the number of cpd declined by 50%.[5] We did not measure carbon monoxide (CO) in this study but others have found a reduction of 30% in this context, suggesting that compensatory smoking is not a significant issue.[5] It has been proposed that smokers regulate their smoking behavior to obtain a constant characteristic nicotine level, for example by increasing smoke intake when the nicotine supply is reduced or decreasing smoke intake when extra nicotine is received from another route. [8] However, mixed results have been obtained when this model has been tested. Foulds et al [9] for example, in a randomized double blind crossover trial of nicotine and placebo patches with *ad libitum *smoking, reported no fall in cpd but a 14% drop in CO together with less satisfaction from cigarettes and fewer and weaker urges to smoke. Our study, however, supports the notion of down-regulation of nicotine intake. Studies examining the effect of nicotine gum on ad-lib smoking have found an inhibitory effect [10] but this may be dose-related.[11] Using nicotine gum may of itself result in a reduction in cigarette smoke intake simply because one cannot smoke while chewing gum. The use of intravenous nicotine infusion avoids this problem[12] but the results are not clear-cut as laboratory-based environments in which such studies are conducted lack the usual cues to smoking.

Fourthly, the two-week pre-quit period appears to a reasonably acceptable pre-quitting period, and likely to be sufficient for effectiveness, based on the experience of Schuurmans et al.[3]

Finally, several themes emerged from the analysis of participant's diaries that shed light on possible mechanisms for any effect, if indeed one exists: an increasing sense of personal control over smoking; and a reduction in the stress associated with changing smoking and cigarette purchasing behavior compared with previous quit attempts.

## Conclusion

Our findings suggest that a short period of around two weeks concomitant smoking and NRT patches or gum at normally prescribed doses may maintain nicotine at high but safe and well-tolerated levels sufficient to minimize cravings. This may facilitate a gradual reduction in smoking through down-regulation. We hypothesize that this, together with a growing sense of personal control over smoking behaviors, may facilitate a more successful and sustained transition to quitting than the usual practice of suddenly stopping the main source of nicotine (i.e. cigarettes) on Quit Day. A large randomized trial is planned to address this key question. The trial will also enquire in detail about smoking activity and NRT use over the pre-quit and immediate post-quit period to further illuminate the various mechanisms that underpin the changes and experiences of smokers undertaking this approach.

## Competing interests

The authors declare that they have no competing interests.
